# Occurrence of new neurons in the piriform cortex

**DOI:** 10.3389/fnana.2014.00167

**Published:** 2015-01-21

**Authors:** Ti-Fei Yuan, Yu-Xiang Liang, Kwok-Fai So

**Affiliations:** ^1^School of Psychology, Nanjing Normal UniversityNanjing, China; ^2^Department of Ophthalmology, The University of Hong KongHong Kong, China; ^3^Department of Anatomy, Li Ka Shing Faculty of Medicine, The University of Hong Kong, Hong KongChina; ^4^The State Key Laboratory of Brain and Cognitive Sciences, The University of Hong KongHong Kong, China; ^5^GHM Institute of CNS Regeneration, Jinan UniversityGuangzhou, China

**Keywords:** adult neurogenesis, piriform cortex, epilepsy, degeneration, Alzheimer’s disease, Parkinson’s disease, olfaction

## Abstract

Adult neurogenesis has been well studied in hippocampus and subventricular zone (SVZ); while this is much less appreciated in other brain regions, including amygdala, hypothalamus, and piriform cortex (PC). The present review aims at summarizing recent advances on the occurrence of new neurons in the PC, their potential origin, and migration route from the SVZ. We further discuss the relevant implications in olfactory dysfunction accompanying the neurodegenerative diseases.

## INTRODUCTION

Adult neurogenesis was first characterized in rodents in the 1960s (Altman and Das, 1965; Altman, 1969), but it was not until the 1990s, when series of studies noted the new neuron addition to adult song bird brain during reproductive season (Goldman and Nottebohm, 1983; Alvarez-Buylla, 1992) that adult neurogenesis began to receive increasing attention. Following data further reported evidences of adult neurogenesis in most rodents, primates, and humans (Gould, 2007). Currently, the idea of new neuron formation in adulthood has been extensively studied and is generally acknowledged.

In rodents, the dentate gyrus (DG) of the hippocampus and the subventricular zone (SVZ) are regarded as the most prominent neurogenic niches in adulthood. The DG neurogenesis is considered important for a series of hippocampus-dependent cognitive functions (Clelland et al., 2009; Sahay et al., 2011), as well as the regulation of emotional response to antidepressants (Sahay and Hen, 2007). Neurogenesis in SVZ is considered to be involved in the fine modulation of olfaction, repair of cortical injuries, and defense of the viral spreading from the central olfactory pathway (Loseva et al., 2009; Yuan, 2010; Lepousez et al., 2013; Gheusi and Lledo, 2014; Magnusson et al., 2014). In addition, there are anatomical sites such as hypothalamus, amygdala, olfactory tubercle, and piriform cortex (PC) with reports of adult neurogenesis (Bernier et al., 2002; Loseva et al., 2009; Pierce and Xu, 2010; Yuan and Arias-Carrion, 2011; Feliciano and Bordey, 2013; Yuan et al., 2014). The present review aims at summarizing recent advances investigating the adult neurogenesis in PC, in terms of origin of new neurons, migration route, potential functions, and implications in therapies against certain neurological disorders.

## ORGANIZATION AND CONNECTIVITY OF PIRIFORM CORTEX

Piriform cortex is a three-layer paleocortex. The first layer of PC receives projections from olfactory bulb through the lateral olfactory tract (LOT; Shipley and Ennis, 1996), while the second and the third layers are composed of principal pyramidal neurons that receive excitatory inputs from the LOT, as well as local interneurons that strongly inhibit these pyramidal neurons (Poo and Isaacson, 2009). In addition, PC pyramidal neurons receive excitatory inputs from connected cortical regions or other pyramidal neurons, forming the association fiber (ASSN) pathway.

**FIGURE 1:**
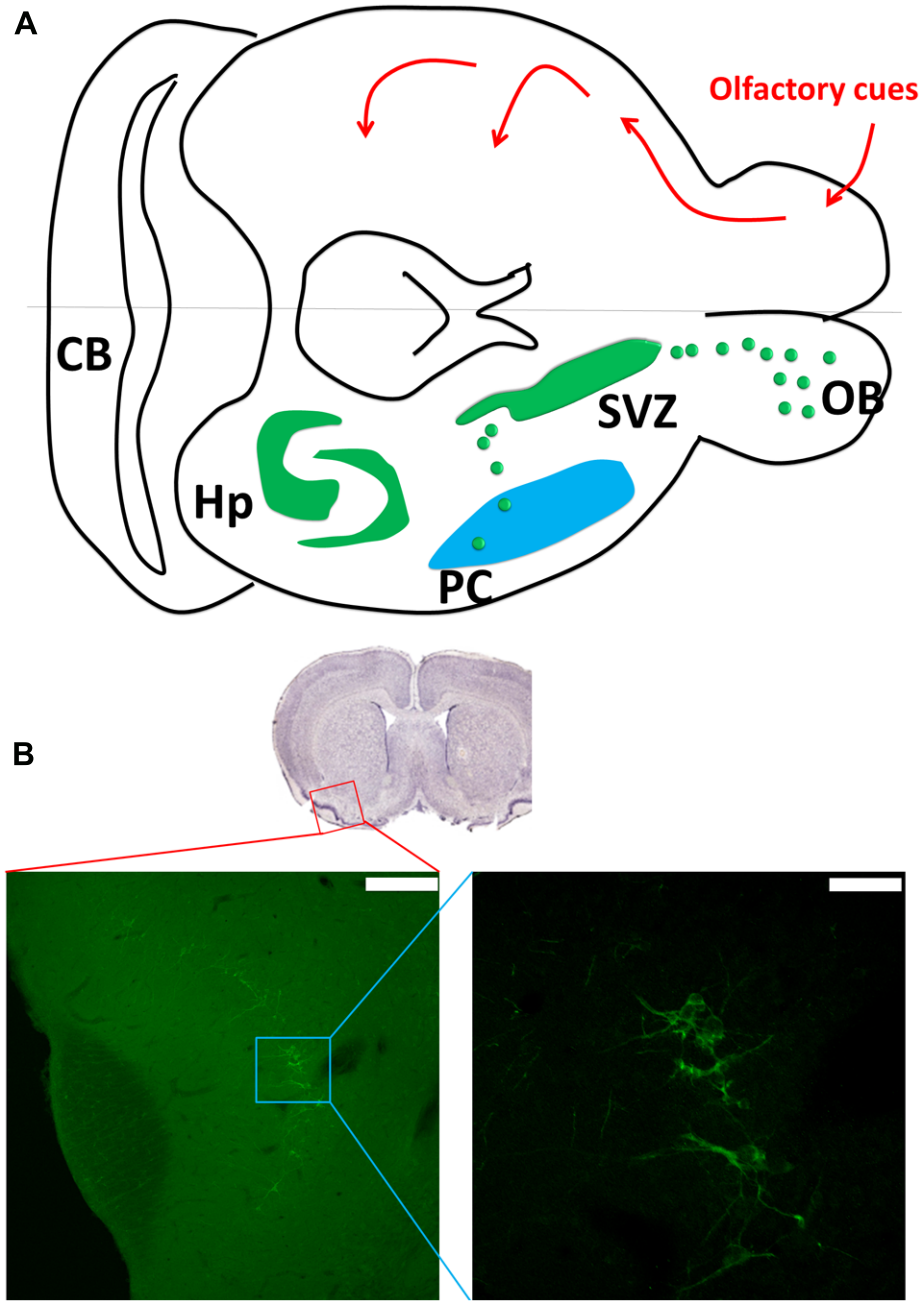
**Piriform cortex (PC) neurogenesis. (A)** The upper part shows the olfactory sensory pathway. The olfactory bulb transmits the signal to the PC as the first relay, and then to other connected regions such as hypothalamus, entorhinal cortex, and amygdala. The lower part of the figure shows the neurogenic sites including the hippocampus and subventricular zone (SVZ). The SVZ gives birth to new neurons in the olfactory bulb and PC through rostral and caudal migration streams. (**A** was modified from Klempin et al., 2011). **(B)** The doublecortin (DCX) staining in the PC (coronal section). Scale bar: left: 200 μm, right: 50 μm.

The LOT-PC pathway is responsible for olfactory information transfer (Uchida et al., 2013). The odors are sparsely coded in pyramidal neurons in PC; through LOT-PC pathway the coded signals can be transmitted to the neocortex, hypothalamus, thalamus, and entorhinal cortex (Shipley and Ennis, 1996; Sullivan and Dryer, 1996). In addition, the PC receives heavy projections from amygdaloid complex (Majak et al., 2004), which plays important roles in amygdala kindling model of epilepsy (Loscher and Ebert, 1996). Pyramidal neurons in the PC show strong associational connections with many neighboring regions that project back to the PC (Johnson et al., 2000). The exact functions of this associational connectivity are yet to be investigated (**Figure 1**).

## OCCURRENCE OF NEW NEURONS IN THE PIRIFORM CORTEX

### EVIDENCE

Most PC neurons are generated during embryonic day 14–18 (E14–E18; Bayer, 1986). In the early 2000s, it was found that PC contains neurons expressing immature neuronal markers, such as doublecortin (DCX) and the polysialylated form of the neural cell adhesion molecule (PSA-NCAM; Nacher et al., 2001, 2002). It was also reported that after amygdala kindling for epilepsy induction, nestin expression was increased in the PC (Nakagawa et al., 2004).

The occurrence of new neurons in the PC was further confirmed by 5-bromo-2-deoxyuridine (BrdU) labeling studies in both rodents and primates (Bernier et al., 2002; Pekcec et al., 2006). These studies showed that the newly generated neurons could become mature (expression of mature neuronal marker, e.g., NeuN). Furthermore, it was proposed that neurogenesis in the PC is not a local phenomenon but rather a migration from the ventricular zone (Shapiro et al., 2007a), DCX-BrdU double-positive cells appear in the PC several days after Brdu labeling, not earlier, suggesting that these cells originated elsewhere and migrated to the PC. In addition, the study of DiI labeling of SVZ neural precursors implied a caudal-ventral migratory stream linking ventricle zone to the PC (Shapiro et al., 2007a).

### COMPARISONS WITH THE HIPPOCAMPUS/SVZ

Interestingly, the DCX-expressing cells in the PC seem to be different from new neurons in the olfactory bulb and hippocampus. Morphologically, they exhibit different dendrite arborization and orientation in comparison to new granule cells in the hippocampus and olfactory bulb with distinct morphologies, including some semilunar pyramidal neurons and neurogliaform cells (Shapiro et al., 2007a; Klempin et al., 2011). In distribution, these new neurons are not organized into one specific layer of the PC, but are sparsely distributed in both layer II and III in the PC.

The functionality of these new neurons shows peculiar features. For instance, whole cell patch clamp recording found that DCX-positive cells in the PC exhibit electrophysiological characteristics of mature neurons, such as large sodium conductance, frequent spikes, and spontaneous post-synaptic currents, which are completely different from those of DG DCX-positive cells (Klempin et al., 2011). It is possible that these DCX positive cells in PC maintain enhanced plasticity even after maturation of other characteristics.

### FATE

It is unclear for how long these DCX/PSA-NCAM-positive cells will maintain their immature state. Lineage tracing through DCX-GFP mice found that some of the DCX-positive cells also express markers of cortical interneurons, such as parvalbumin and calretinin (Bedard and Parent, 2004; Klempin et al., 2011), which is consistent with their origin from the SVZ; while another study found that these new cells could form principal cells, namely, pyramidal neurons in the PC (Guo et al., 2010).

The BrdU labeling study showed that there are many non-BrdU-labeled DCX positive cells in the PC, even after pulsed labeling (Shapiro et al., 2007b), this might indicate a low proliferation rate of neural stem/progenitor cells generating new neurons for the PC. Alternatively, it is possible that these cells maintain the expression of plastic genes as immature neurons, and participate in olfactory signal coding – given that they have already received some spontaneous synaptic currents (Klempin et al., 2011).

## POTENTIAL ROLES

The addition of new neurons, especially interneurons, to existing neural circuits leads to significant changes (Cameron and Dayer, 2008; Lepousez et al., 2013). The complexity of intracortical circuits is increased with the enhancement of neuronal plasticity in the PC, and the existing synaptic connections can also be modulated. On the other hand, it is still unknown if these new neurons can turn into principal cells in the PC. In one study, DCX-positive cells in adult PC co-expressing cellular markers (NG2, PDGFR-alpha) of oligodendroglial progenitor cells (OPCs) were finally found to exhibit principal cell morphology, showing glutamatergic neuron identity (Guo et al., 2010). Thus, it will be interesting to analyze the synaptic connections of these cells in future studies, in order to understand their functional implications in olfactory information processing.

It is possible that PC neurogenesis responds to local injury signals or olfactory bulbectomy. Given the theory of ventricle origin of PC new neurons (Shapiro et al., 2007a), this is consistent with other findings showing that adult born new neurons can migrate to and repair the ischemic cortex (Ruan et al., 2014), for instance. A recent study found that upon stroke injury, local neurogenesis in the striatum can result from astrocytes (Magnusson et al., 2014). It will therefore be interesting to examine if local neurogenesis in PC exists in pathological conditions, such as epilepsy and other kinds of injury.

Chronic stress decreases PC neurogenesis (Nacher et al., 2004), on line with the findings in the hippocampus and SVZ. This may partly explain the olfactory system dysfunction in major depression (Yuan and Slotnick, 2014). Recent studies have also highlighted the loss of olfaction during the pathogenesis of neurodegenerative diseases, such as Alzheimer’s disease (AD) and Parkinson’s disease (Doty, 2012; Li et al., 2013). Interestingly, in AD patients the deficits in odor quality coding originated from dysfunction in the PC (Li et al., 2010). Given the important roles of new neurons from a computational perspective, it will be useful to investigate the potential changes of PC neurogenesis in animal models of AD/PD and human patient postmortem brain samples.

## TARGETING NEUROGENESIS IN THE PIRIFORM CORTEX

It is known that direct administration of neurotrophic factors [Ciliary neurotrophic factor (CNTF) and brain-derived neurotrophic factor (BDNF)] into the ventricle robustly boosts the neurogenesis at the SVZ and the hypothalamus (Pencea et al., 2001; Kokoeva et al., 2005). Conceivably, this will also enhance the PC neurogenesis through migration. In addition, the well-known intranasal route of trophic factor application could result in better targeting of the olfactory cortex (Sood et al., 2014). There are also numerous pharmacological agents that could block or promote neurogenesis, which have been well studied in hippocampus and SVZ neurogenesis signaling pathways (Suh et al., 2009; Christian et al., 2013; Lepousez et al., 2013). For instance, NMDA receptor antagonism leads to increased PSA-NCAM expression in the PC (Nacher et al., 2002).

Sensory input is critical for neurogenesis in the SVZ and rostral migratory stream (RMS), as well as the fate determination of new neurons in the olfactory bulb (Alonso et al., 2008; Sawada et al., 2011). It will be also necessary to understand if odor experience can modulate the PC neurogenesis as well. Indeed, olfactory enrichment could enhance the PC neurogenesis (Shapiro et al., 2007b). Furthermore, it will be interesting to examine if electroconvulsive therapy, deep brain stimulation or non-invasive brain stimulation approaches (e.g., rTMS) are able to modulate PC neurogenesis.

## SUMMARY

In summary, adult neurogenesis in olfactory cortical regions, especially the PC is much less appreciated in compared to hippocampus and SVZ neurogenesis. However, it may have important functional implications in olfactory representation in the PC. Disruption of adult neurogenesis is found in epilepsy and neurodegenerative diseases; it will be interesting to examine if the loss of olfaction is associated with disrupted PC neurogenesis in brain diseases.

## Conflict of Interest Statement

The authors declare that the research was conducted in the absence of any commercial or financial relationships that could be construed as a potential conflict of interest.
